# Roseovarius phycicola sp. nov. and Roseovarius rhodophyticola sp. nov., isolated from marine red algae

**DOI:** 10.1099/ijsem.0.006574

**Published:** 2024-11-11

**Authors:** Min Woo Lee, Ju Hye Baek, Jeong Min Kim, Hülya Bayburt, Byeong Jun Choi, Baolei Jia, Che Ok Jeon

**Affiliations:** 1 Department of Life Science, Chung-Ang University, Seoul 06974, Republic of Korea; 2 Xianghu Laboratory, Hangzhou 311231, PR China

**Keywords:** marine red algae, new taxa, *Pseudomonadota*, *Roseovarius phycicola*, *Roseovarius rhodophyticola*

## Abstract

Two Gram-stain-negative, strictly aerobic, non-motile, rod-shaped bacteria, designated as strains S88^T^ and W115^T^, exhibiting catalase- and oxidase-positive reactions, were isolated from marine red algae in South Korea. Strain S88^T^ exhibited growth at 20–30 °C, pH 6.0–9.0 and 2.0–5.0% (w/v) NaCl, while strain W115^T^ grew at 20–30 °C, pH 7.0–9.0 and 2.0–5.0% (w/v) NaCl. Strain S88^T^ contained summed feature 8 (C_18 : 1_* ω*7*c* and/or C_18 : 1_* ω*6*c*), C_16 : 0_ and C_16 : 0_ 2-OH as major fatty acids (>5%), with major polar lipids being phosphatidylglycerol, phosphatidylcholine, an unidentified phospholipid, an unidentified aminolipid and two unidentified lipids. Strain W115^T^ contained summed feature 8 (C_18 : 1_* ω*7*c* and/or C_18 : 1_* ω*6*c*) and C_16 : 0_ as major fatty acids (>5%), with major polar lipids including phosphatidylglycerol, phosphatidylcholine, an unidentified phospholipid, an unidentified aminolipid and unidentified lipids. Ubiquinone-10 was the sole respiratory quinone in both strains, and the genomic DNA G+C contents were 57.0% for strain S88^T^ and 56.5% for strain W115^T^. Despite 99.86% 16S rRNA gene sequence similarity, strains S88^T^ and W115^T^ shared 88.8% average nucleotide identity (ANI) and 36.8% digital DNA–DNA hybridization (dDDH) value, indicating different species. Phylogenetic and phylogenomic analyses based on 16S rRNA gene and genome sequences, respectively, revealed that strains S88^T^ and W115^T^ formed a phylogenetic lineage within the genus *Roseovarius*. ANI and dDDH values of both strains with other type strains were less than 73.7 and 20.3%, respectively, confirming that they represent novel species. Based on phenotypic, chemotaxonomic and molecular characteristics, strains S88^T^ and W115^T^ represent two novel species of the genus *Roseovarius*, for which the names *Roseovarius phycicola* sp. nov. (S88^T^ =KACC 23423^T^ =JCM 36647^T^) and *Roseovarius rhodophyticola* sp. nov. (W115^T^ =KACC 23690^T^ =JCM 36651^T^) are proposed, respectively.

## Introduction

The genus *Roseovarius*, belonging to the phylum *Pseudomonadota*, was first proposed by Labrenz *et al*. [1] in 1999 to describe *Roseovarius tolerans* as the type species. A recent study based on whole-genome phylogenetic and genotypic analyses showed that the genus *Roseovarius* is paraphyletic and belongs to the family *Roseobacteraceae*, which was separated from the family *Rhodobacteraceae* into a distinct family [2]. As of September 2024, this genus includes 35 validly published and 5 invalidly published species (https://lpsn.dsmz.de/genus/roseovarius) [3], which have primarily been isolated from marine environments such as estuary sediment [4], tidal flat [5,7], seawater [8,9], coastal seawater [10,11], saline lakes [12,13] and marine organisms [14,16]. Members of the genus *Roseovarius* are physiologically diverse, even with some species displaying aerobic anoxygenic phototrophy, dimethylsulfoniopropionate and aromatic hydrocarbon degradation and capability for bioleaching gold [1,17,20]. The genus *Roseovarius* is typically Gram-stain-negative, non-spore-forming, strictly aerobic, NaCl-requiring chemoheteroorganotrophs that are ovoid or rod-shaped and exhibit catalase- and oxidase-positive activities [4,9]. *Roseovarius* cells contain ubiquinone-10 (Q-10) as the predominant respiratory quinone, with summed feature 8 (C_18 : 1_* ω*7*c* and/or C_18 : 1_* ω*6*c*) and C_16 : 0_ as major fatty acids, and phosphatidylcholine (PC) and phosphatidylglycerol (PG) as major polar lipids [4,9]. During our research on interactions between marine algae and bacteria, we have isolated numerous novel bacteria from the phycosphere of marine algae [21,23]. In this study, we isolated two potentially novel strains affiliated with the genus *Roseovarius* from marine algae and characterized their taxonomic properties using a polyphasic approach.

## Strain isolation

Strains S88^T^ and W115^T^ were isolated from marine red algae *Lomentaria hakodatensis* and *Schizymenia dubyi*, respectively, collected from Nogu Port (34° 45′ 24′ N, 128° 03′ 14′ E) and Guryepo Beach (36° 53′ 29′ N, 126° 11′ 53′ E) in South Korea, as previously described with some modifications [24]. Briefly, the collected marine algae were washed by agitating them in sterilized artificial seawater (ASW) (20 g NaCl, 2.9 g MgSO_4_, 4.53 g MgCl_2_∙6H_2_O, 0.64 g KCl and 1.75 g CaCl_2_∙2H_2_O per litre). The washed marine algae were mechanically homogenized using a T10 basic homogenizer (IKA, Germany) for 1 min and serially diluted in ASW. Aliquots (100 µl) of each dilution were spread on marine agar (MA) (MBcell, South Korea) and aerobically incubated at 25 °C for 3 days. The 16S rRNA genes of colonies grown on MA were PCR-amplified using the universal primers 27F (5′-AGA GTT TGA TCM TGG CTC AG-3′) and 1492R (5′-TAC GGY TAC CTT GTT ACG ACT T-3′) [24]. The PCR products were double-digested with *Hae*III and *Hha*I, then analysed by 2% (w/v) agarose electrophoresis. Representative PCR amplicons showing distinct fragment patterns were partially sequenced using the primer 340F (5′-CCT ACG GGA GGC AGC AG-3′) [24] at Macrogen (South Korea), and the partial 16S rRNA gene sequences were compared with those of all type strains of validly published species on the EzBioCloud server (http://www.ezbiocloud.net/) [25]. From the analysis, two potential novel strains (S88^T^ and W115^T^) belonging to the genus *Roseovarius* were selected for further phenotypic and phylogenetic analyses. These strains were routinely cultured aerobically on MA at 25 °C for 3 days and preserved at –80 °C in marine broth (MB) (MBcell) supplemented with 15 % (v/v) glycerol.

## Phylogeny based on 16S rRNA gene sequences

The 16S rRNA genes of strains S88^T^ and W115^T^, amplified using the 27F and 1492R primers, were further sequenced with the universal 16S rRNA primers 518R (5′-ATT ACC GCG GCT GCT GG-3′) and 805F (5′-GAT TAG ATA CCC TGG TAG TC-3′) [24]. Nearly complete 16S rRNA gene sequences were obtained for strain S88^T^ (1417 nucleotides) and strain W115^T^ (1406 nucleotides) by assembling the sequences from the 340F, 518R and 805F primers. The similarities among the 16S rRNA gene sequences of strains S88^T^ and W115^T^ and their closely related type strains were calculated using EzBioCloud. The 16S rRNA gene sequences of strains S88^T^ and W115^T^ and closely related type strains were aligned using Infernal (ver. 1.1.4) with the covariance model of Rfam family RF00177 [26]. Phylogenetic trees were constructed based on the neighbour-joining (NJ), maximum-parsimony (MP), and maximum-likelihood (ML) algorithms, with bootstrap values (1000 replications), using mega11 software [27]. The Kimura two-parameter model, the nearest-neighbour-interchange heuristic search method and the pairwise deletion options were used in constructing the NJ, MP and ML trees, respectively.

**Fig. 1.**
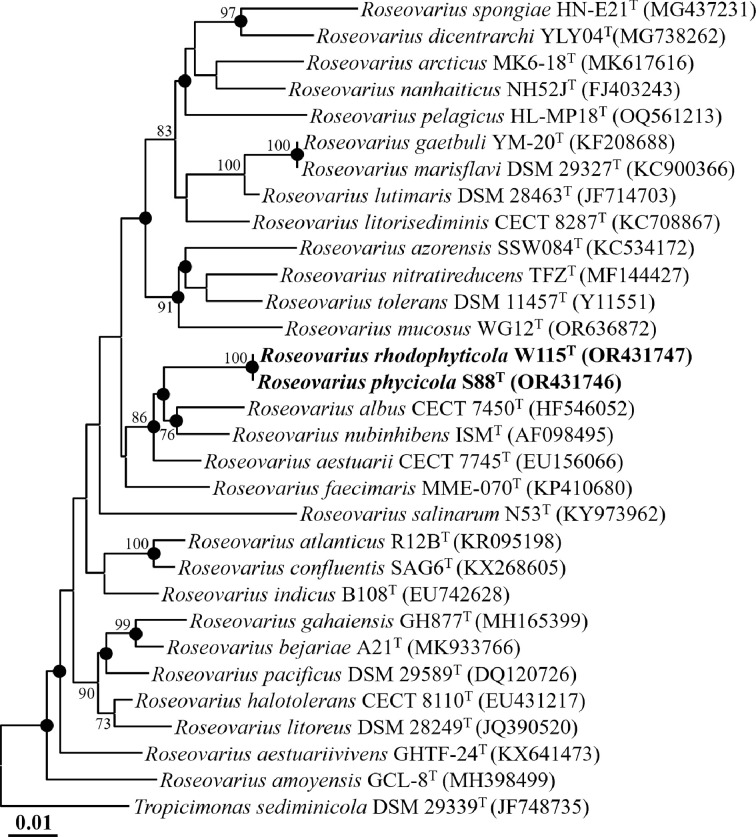
NJ tree showing the phylogenetic relationships between strains S88^T^ and W115^T^ and closely related taxa, based on 16S rRNA gene sequences. Bootstrap values exceeding 70% are indicated on the nodes as percentages from 1000 replicates. Filled circles (●) denote nodes that were also retrieved in the trees constructed using the ML and MP algorithms. *Tropicimonas sediminicola* DSM 29339^T^ (JF748735) was employed as an outgroup. Bar, 0.01 substitutions per nucleotide.

The 16S rRNA gene sequences of strains S88^T^ and W115^T^ were found to be 99.86 % identical. Comparative analysis of these sequences with those of other type strains revealed that strains S88^T^ and W115^T^ were most closely related to *Roseovarius nubinhibens* ISM^T^ (97.12 and 97.05% similarity, respectively), *Roseovarius albus* CECT 7450^T^ (96.68 and 96.61% similarity, respectively), *Roseovarius faecimaris* MME-070^T^ (96.25 and 96.18% similarity, respectively), *Roseovarius aestuariivivens* GHTF-24^T^ (96.03 and 95.96% similarity, respectively) and *Roseovarius halotolerans* DSM 29507^T^ (95.53% similarity for both). Phylogenetic analysis based on 16S rRNA gene sequences using the NJ algorithm showed that strains S88^T^ and W115^T^ formed a distinct phylogenetic lineage within the genus *Roseovarius* (Fig. 1). This affiliation was also supported by phylogenetic trees reconstructed using the ML and MP algorithms (Fig. S1, available in the online Supplementary Material). These comparative and phylogenetic analyses suggest that strains S88^T^ and W115^T^ belong to the genus *Roseovarius*. Based on the 16S rRNA gene sequence similarities and phylogenetic trees, *R. nubinhibens* DSM 15170^T^, *R. albus* KCTC 22653^T^, *R. faecimaris* KCCM 43142^T^, *R. aestuariivivens* KCTC 52454^T^ and *R. halotolerans* KCTC 22224^T^ were selected for comparisons of fatty acid compositions, as well as genomic and phenotypic characteristics.

## Whole-genome sequencing and genome-based phylogeny

The genomic DNA of strains S88^T^ and W115^T^ was extracted from cells cultured in MB using the Wizard Genomic DNA Purification Kit (Promega, USA), following the manufacturer’s instructions. The extracted genomic DNA was sequenced in-house using the Oxford Nanopore MinION (ONT, UK). The resulting sequencing reads were *de novo* assembled using Flye (ver. 2.9.1) [28]. The assembled genomes were quality-checked for completeness and contamination rates using CheckM2 software (ver. 1.0.2) [29].

The *de novo* assembly of MinION sequencing data from strains S88^T^ and W115^T^, with average genome coverages of approximately 58.0× and 86.0×, respectively, yielded complete genomes of 3637 kb and 3717 kb. The genome of strain S88^T^ consisted of 2 contigs: a 3536 kb chromosome and a 101 kb plasmid. The genome of strain W115^T^ also comprised two contigs: a 3613 kb chromosome and a 104 kb plasmid. The 16S rRNA gene sequences in the genomes of strains S88^T^ and W115^T^ (1466 nucleotides for both strains) included sequences identical to those obtained by PCR-based sequencing. The completeness and contamination rates were 99.38 and 0% for strain S88^T^ and 99.56 and 0.51% for strain W115^T^, respectively, meeting the criteria for high-quality genomes (≥90% completeness and ≤10% contamination) [29].

A phylogenomic analysis of strains S88^T^ and W115^T^, along with closely related type strains, was conducted using the Genome Taxonomy Database Toolkit (GTDB-Tk), based on the concatenated protein sequences of 120 ubiquitous single-copy marker genes (bac120 marker set) [30]. The alignment of these concatenated protein sequences and the reconstruction of the phylogenomic ML tree, including bootstrap values from 1000 replications, were performed using mega11 software. Average nucleotide identity (ANI) and digital DNA–DNA hybridization (dDDH) values among strains S88^T^ and W115^T^, as well as other closely related type strains, were calculated using the Orthologous ANI Tool online (ver. 0.93.1; www.ezbiocloud.net/tools/orthoani) [31] and the Genome-to-Genome Distance Calculator (GGDC 3.0; https://ggdc.dsmz.de/ggdc.php) with formula 2 [32], respectively.

**Fig. 2.**
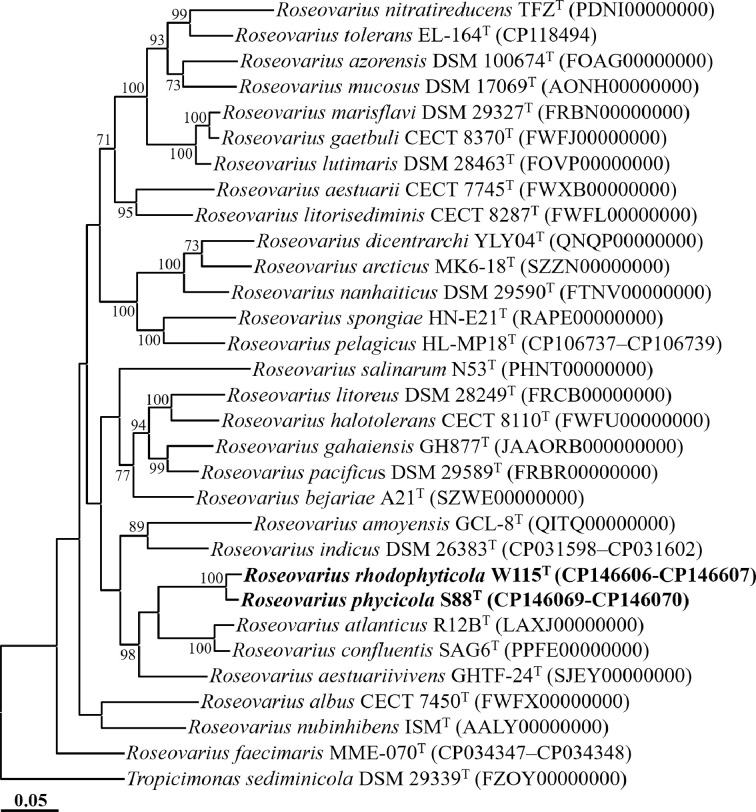
ML phylogenomic tree showing the phylogenetic relationships between strains S88^T^ and W115^T^ and closely related taxa, based on the concatenation of 120-bacterial protein marker set (bac120 marker set) of GTDB-Tk. Bootstrap values exceeding 70% are indicated on the nodes as percentages from 1000 replicates. *Tropicimonas sediminicola* DSM 29339^T^ (FZOY00000000) was employed as an outgroup.The scale bar represents 0.05 changes per amino acid position.

The genome-based phylogenomic tree revealed that strains S88^T^ and W115^T^ formed distinct phyletic lineages within the genus *Roseovarius* (Fig. 2), confirming their classification within the genus, as suggested by 16S rRNA gene sequence analyses. Strains S88^T^ and W115^T^ displayed ANI values of 88.8% and dDDH values of 36.8%, both falling below the thresholds for prokaryotic species delineation (ANI, ~95%; dDDH, 70%) [33], indicating that they represent distinct species within the genus *Roseovarius* despite their nearly identical 16S rRNA gene sequences. Additionally, the ANI and dDDH values between strains S88^T^ and W115^T^ and other *Roseovarius* species were below 73.7 and 20.3%, respectively (Table S1), further supporting their status as separate species. The results from the phylogenomic analysis and genome relatedness assessments strongly support the conclusion that strains S88^T^ and W115^T^ represent distinct novel species within the genus *Roseovarius*.

## Genomic features and algal symbiosis and marine environment-associated genes

The whole-genome sequences of strains S88^T^ and W115^T^ were submitted to GenBank and annotated using the NCBI Prokaryotic Genome Annotation Pipeline. The genome of strain S88^T^ was predicted to harbour 3673 genes (3571 in the chromosome and 102 in the plasmid), including 3616 protein-coding sequences (3514 in the chromosome and 102 in the plasmid), one rRNA operon (16S, 23S, 5S) and 51 tRNA genes. Similarly, the genome of strain W115^T^ contained 3789 genes (3675 in the chromosome and 114 in the plasmid), including 3740 protein-coding sequences (3634 in the chromosome and 106 in the plasmid), 1 rRNA operon (16S, 23S, 5S) and 43 tRNA genes (Table 1). These genomic features, including genome size, total gene numbers, protein-coding genes and tRNA gene numbers, were comparable to those of closely related *Roseovarius* species. Additionally, the G+C contents of the genomic DNA of strains S88^T^ and W115^T^ were 57.0 and 56.5%, respectively, slightly lower than those of other closely related *Roseovarius* species (Table 1).

**Table 1.** General genomic features of strains S88^T^ and W115^T^ and closely related type strains of the genus *Roseovarius* Taxa: 1, strain S88^T^ (CP146069-CP146070); 2, strain W115^T^ (CP146606-CP146607); 3, *R. nubinhibens* ISM^T^ (AALY00000000); 4, *R. albus* CECT 7450^T^ (FWFX00000000); 5, *R. faecimaris* MME-070^T^ (CP034347-CP034348); 6, *R. aestuariivivens* GHTF-24^T^ (SJEY00000000); 7, *R. halotolerans* DSM 29507^T^ (RBXI00000000). The genomes of strains S88^T^ and W115^T^ were sequenced in this study.

Feature*	1	2	3	4	5	6	7
Genome status (no. of contigs)†	C (2)	C (2)	D (10)	D (39)	C (2)	D (39)	D (11)
Total genome size (kb)	3637	3717	3676	4288	3935	3942	3718
G+C content (%)	57.0	56.5	64.0	54.5	62.5	62.0	64.0
No. of total genes	3673	3789	3561	4151	3849	3838	3692
No. of protein-coding sequences	3616	3740	3506	4102	3798	3789	3622
No. of total RNA	57	49	55	49	51	49	48
No. of tRNA	51	43	46	43	45	43	42
No. of rRNA (16S, 23S, 5S) operon	1	1	2	1	1	1	1
No. of pseudogenes	87	136	18	26	11	32	22
No. of total CAZy† genes	63	59	59	67	68	56	55
Auxiliary activities	9	6	11	8	8	5	9
Carbohydrate-binding module	1	2	1	4	4	1	1
Carbohydrate esterase	2	2	1	3	4	1	2
Glycoside hydrolase	15	14	14	12	14	13	15
Glycosyltransferase	36	35	32	40	38	36	28
Polysaccharide lyase	0	0	0	0	0	0	0

* The genomic features, excluding CAZy genes, were analyzedanalysed using the NCBI pProkaryotic gGenome aAnnotation pPipeline (www.ncbi.nlm.nih.gov/genome/annotation_prok/).

† C, complete; D, draft; CAZy, carbohydrate-active enzyme.

Algae are primarily composed of polysaccharides, which are essential components of their extracellular matrices, cell walls and storage compounds. Consequently, the ability to degrade various algal polysaccharides may be significant among heterotrophic bacteria associated with marine algae. To predict the algal polysaccharide-degrading abilities of strains S88^T^ and W115^T^, as well as closely related *Roseovarius* strains, the carbohydrate-active enzymes (CAZys) were analysed by submitting their protein sequences to the dbCAN3 meta server (https://bcb.unl.edu/dbCAN2/blast.php) [34]. The genomes of strains S88^T^ and W115^T^ were predicted to harbour 63 and 59 genes encoding various CAZys, respectively, similar to other closely related *Roseovarius* strains (Table 1). Despite being isolated from marine algae, strains S88^T^ and W115^T^, as well as closely related *Roseovarius* strains, have fewer CAZy genes and lack genes encoding polysaccharide lyases, compared to other bacteria isolated from marine algae [21,23]. This suggests a potentially limited ability of these strains to degrade algal polysaccharides and highlights the need for further experimental research to explore other interactions that strains S88^T^ and W115^T^ may have with their marine algal hosts.

Bacteria residing in the phycosphere of algae can affect their algal hosts through diverse metabolic interactions, including the production of compounds like vitamins and nutrients that promote algal growth [35]. Genomic analysis revealed that strains S88^T^ and W115^T^ possess all the essential genes for cobalamin biosynthesis from glycine (*hemABC*, *cysG*, *cobAIGJMKLH*, *cobB*, *cobNST*, *cobO* and *cbiBP*), except for *hemD* and *cobF*. Additionally, both strains have the genes necessary for riboflavin synthesis from ribulose 5-phosphate (*ribBEH*). These vitamin biosynthetic capabilities in strains S88^T^ and W115^T^ suggest that they may have beneficial effects on the growth of their algal hosts [36].

Marine microbes use various strategies to cope with environmental stresses, such as osmotic stress caused by high salinity [37]. In high-salinity environments, biological cells must maintain osmotic balance. Marine bacteria are known to produce compatible solutes to achieve this balance [38]. Strains S88^T^ and W115^T^ have genes for synthesizing the compatible solute glycine betaine (*gbsA* and *gbsB*) from choline, which encode glycine betaine aldehyde dehydrogenase (GsbA) and type-III alcohol dehydrogenase (GbsB), respectively. Therefore, strains S88^T^ and W115^T^ are expected to exhibit tolerance and adaptation to salinity stress in marine habitats through the biosynthesis of glycine betaine.

## Morphological and physiological characteristics

The growth of strains S88^T^ and W115^T^ was evaluated on various agar media (all from MBcell), including MA, Reasoner’s 2A (R2A) agar, Luria-Bertani (LB) agar, tryptic soy agar (TSA) and nutrient agar (NA) with 2% (w/v) NaCl, at 25 °C for 3 days. Additionally, growth was tested on MA at different temperatures (10–45 °C in 5 °C intervals) and at varying pH values (4.0 to 11.0 in 1.0-unit intervals) in MB at 25 °C for 3 days. MB media with various pH levels were prepared using sodium citrate (pH 4.0–5.0), sodium phosphate (pH 6.0–8.0) and sodium carbonate-bicarbonate (pH 9.0–11.0) buffers, with pH adjustments made after autoclaving if necessary. Growth at various NaCl concentrations was evaluated for 3 days at 25 °C in MB medium (ranging from 0 to 10% in 1.0% intervals, w/v), prepared according to the laboratory MB medium composition. Anaerobic growth was assessed on MA and MA supplemented with 0.1% (w/v) KNO_3_ after 21 days of incubation at 25 °C using the GasPak Plus system (BBL, USA). All physiological and biochemical tests were conducted using cells grown on MA at 25 °C for 3 days. Cellular morphology and motility of strains S88^T^ and W115^T^ were examined under a phase-contrast microscope (Zeiss Axio Scope. A1; Carl Zeiss, Germany). For detailed examinations of morphology and flagella, cells of strains S88^T^ and W115^T^ were affixed to formvar-coated copper grids, negatively stained with 2% (w/v) uranyl acetate (Sigma-Aldrich, USA) for 15 s and observed under a transmission electron microscope (JEM-1010; JEOL, Japan). The motility of strains S88^T^ and W115^T^ was further evaluated in MA containing 0.3% (w/v) agar by inoculating the agar plates by stabbing with the strains and incubating them at 25 °C. Gram staining was conducted using a Gram stain kit (bioMérieux, France) according to the manufacturer’s instructions. Catalase activity was determined by observing oxygen bubble production in a 3% (v/v) aqueous hydrogen peroxide solution (Junsei, Japan), and oxidase activity was evaluated by the oxidation of 1% (w/v) tetramethyl-*p*-phenylenediamine (Merck, USA) [39]. The following phenotypic traits of strains S88^T^ and W115^T^ were compared with reference strains under the same conditions at their optimal temperatures. Hydrolysis of tyrosine, casein, urea, aesculin, starch, gelatin, Tween 20 and Tween 80 was investigated on MA, as previously described [39]. Additional biochemical features were assessed using API 20NE kits from bioMérieux (France), following the manufacturer’s instructions, with cells resuspended in ASW used as the inocula.

Strains S88^T^ and W115^T^ optimally grow on MA but exhibit slow growth on R2A agar, LB agar, TSA and NA containing 2% NaCl. Cells of both strains are Gram-stain-negative rods, lacking flagella, and measuring 0.7–1.0 µm wide and 1.0–1.6 µm long for strain S88^T^, and 0.6–0.7 µm wide and 0.9–2.0 µm long for strain W115^T^ (Fig. S2). Their motility characteristics were confirmed through observations under a phase-contrast microscope and motility tests on 0.3% agar media. Anaerobic growth was not observed for either strain, indicating their strict aerobic nature. Both strains shared many characteristics with reference strains of the genus *Roseovarius*, such as being rod-shaped, non-motile, oxidase and catalase activities, and lacking nitrate reduction ability and glucose fermentation, as well as not hydrolysing casein, tyrosine and starch (Table 2). However, some characteristics, such as colony colour, growth ranges and the hydrolysis of aesculin; Tween 20; Tween 80; and gelatin, differentiated strains S88^T^ and W115^T^ from closely related *Roseovarius* species.

**Table 2.** Differential characteristics between strains S88^T^ and W115^T^ and closely related type strains of the genus *Roseovarius* Taxa: 1, strain S88^T^ (this study); 2, strain W115^T^ (this study); 3, *R. nubinhibens* DSM 15170^T^ [9,18]; 4, *R. albus* KCTC 22653^T^ [8]; 5, *R. faecimaris* KCCM 43142^T^ [6]; 6, *R. aestuariivivens* KCTC 52454^T^ [5]; 7, *R. halotolerans* KCTC 22224^T^ [9]. All strains are positive for the following characteristics: activity* of oxidase and catalase. All strains are negative for the following characteristics: nitrate reduction*; glucose fermentation*; hydrolysis* of casein, tyrosine and starch; assimilation* of capric acid; and indole production. Symbols: +, positive; −, negative; na, no data available.

Characteristics	1	2	3	4	5	6	7
Isolation source	Marine red alga	Marine red alga	Seawater	Seawater	Tidal flat	Tidal flat	Deep seawater
Colony colour	Greyish yellow	Greyish yellow	Cream	Whitish	Cream	Greyish yellow	White to pink
Flagella motility	−	−	+	−	−	−	−
Growth range of:							
Temperature (optimum, °C)	20–30(25)	20–30(25)	10–40(30)	15–28(25)	20–40(25–30)	10–40(30)	10–45(35)
pH (optimum)	6.0–9.0(8.0)	7.0–9.0(8.0)	na	na	7.0–9.0(8.0)	5.5– na(7.0–8.0)	6.0–10.0(7.5)
NaCl (optimum, %)	2.0–5.0(3.0)	2.0–5.0(3.0)	0.5–12.5(2.0–5.0)	1.7–5.0(NA)	1.0–5.0(2.0–3.0)	0–10.0(2.0–3.0)	0.5–20.0(3.0–4.0)
Hydrolysis of:							
Aesculin	+	+	–	–	–	+	+
Tween 20, Tween 80	–	–	–	–	–	+	–
Gelatin	–	–	–	–	+	–	–
Enzyme activity* of:							
Urease	–	–	+	+	–	+	+
*β*-Galactosidase	–	–	–	–	+	–	–
Arginine dihydrolase	–	–	+	+	–	–	–
Assimilation* of:							
d-Glucose	+	–	+	+	+	–	–
l-Arabinose	+	+	+	+	+	+	–
d-Mannose	+	–	+	+	–	+	+
d-Mannitol	–	–	–	+	+	–	+
*N*-Acetylglucosamine	+	–	+	+	–	–	+
d-Maltose	+	+	+	+	+	–	+
Potassium gluconate	+	–	+	+	+	+	–
Adipic acid	+	+	+	+	–	–	–
Malic acid	+	–	+	+	+	+	+
Citric acid	–	+	+	+	–	–	+
Phenylacetic acid	–	+	+	–	–	–	–
Major fatty acids (>5 %)	Summed feature 8, C_16 : 0_, C_16 : 0_ 2-OH	Summed feature 8, C_16 : 0_	Summed feature 8, C_16 : 0_, cyclo-C_19 : 0_* ω8*c	Summed feature 8, C_10 : 0_, C_12 : 0_, C_16 : 0_, C_16 : 0_ 2-OH	Summed feature 8, C_18 : 1_ *ω*7*c* 11-methyl, C_16 : 0_, C_16 : 0_ 2-OH	Summed feature 8, C_16 : 0_	Summed feature 8, C_18 : 1_ *ω*7*c* 11-methyl, cyclo-C_19 : 0_* ω8*c
Major polar lipids	PG, PC, PL, AL, L	PG, PC, PL, AL, L	na	na	PG, PC, DPG, AL, L	PG, PC, PE, DPG	PG, PC, DPG, AL, L

* These data were obtained from this study under the same conditions.

PG, phosphatidylglycerol; PC, phosphatidylcholine; DPG, diphosphatidylglycerol; PE, phosphatidylethanolamine; AL, unidentified aminolipid; L, unidentified lipid.

## Chemotaxonomic characteristics

Respiratory isoprenoid quinones of strains S88^T^ and W115^T^ were extracted from cells cultured in MB for 3 days at 25 °C and analysed using an HPLC system (model LC-20A, Shimadzu, Japan) equipped with a reversed-phase column (250×4.6 mm, Kromasil, Akzo Nobel, Netherlands) and a diode-array detector (SPD-M20A, Shimadzu), with a methanol–isopropanol eluent (2 : 1, v/v) at a flow rate of 1 ml min^−1^, as previously detailed [40]. For cellular fatty acid analysis, strains S88^T^ and W115^T^, along with five reference strains, were cultivated aerobically in MB at their optimal growth temperatures and harvested at the exponential growth phase (OD_600_=0.7–0.8). Fatty acid methyl ester samples were prepared using the standard MIDI protocol (Sherlock Microbial Identification System, version 6.2B), involving saponification, methylation and extraction. These samples were then analysed using a gas chromatograph (Hewlett Packard 6890, USA) and identified with the RTSBA6 database of the Microbial Identification System (Sherlock version 6.0B) [41]. Polar lipids of strains S88^T^ and W115^T^ were analysed using two-dimensional TLC. Cells were harvested during the exponential growth phase, following the procedure described by Minnikin *et al*. [40]. Various reagents were employed to detect different polar lipids: 10 % ethanolic molybdophosphoric acid for total polar lipids, ninhydrin for aminolipids, Dittmer-Lester for phospholipids and *α*-naphthol/sulfuric acid for glycolipids. The presence of PG and PC in strains S88^T^ and W115^T^ was confirmed using standard polar lipid compounds from Sigma-Aldrich (USA).

The respiratory isoprenoid quinone identified in strains S88^T^ and W115^T^ was exclusively Q-10, consistent with other *Roseovarius* species [4,9]. The major cellular fatty acids (>5.0% of the total fatty acids) in strain S88^T^ were summed feature 8 (comprising C_18 : 1_* ω7*c and/or C_18 : 1_* ω6*c), C_16 : 0_ and C_16 : 0_ 2-OH, while those in strain W115^T^ were summed feature 8 and C_16 : 0_ (Table S2). Although the overall fatty acid profiles of strains S88^T^ and W115^T^ were similar to those of closely related *Roseovarius* type strains, there were differences in the proportions of certain fatty acids. For instance, C_16 : 0_ 2-OH, which was abundantly identified in strain S88^T^, *R. albus* and *R. faecimaris*, was not detected or only minimally present in other strains. Regarding polar lipids, both strains S88^T^ and W115^T^ contained PG, PC, an unidentified phospholipid (PL) and an unidentified aminolipid (AL) as major components. Additionally, strain S88^T^ had two unidentified lipids (Ls), and strain W115^T^ had an L (Fig. S3). The presence of PG and PC as major polar lipids in strains S88^T^ and W115^T^ was consistent with other *Roseovarius* species. However, the absence of diphosphatidylglycerol or phosphatidylethanolamine, which are present in other closely related *Roseovarius* species, distinguished these strains from other *Roseovarius* species (Table 2).

## Taxonomic conclusion

The phylogenetic inference, genomic relatedness and phenotypic, biochemical and chemotaxonomic characteristics strongly support that strains S88^T^ and W115^T^ represent two different novel species of the genus *Roseovarius*. Therefore, we propose the names *Roseovarius phycicola* sp. nov. and *Roseovarius rhodophyticola* sp. nov. for novel species, respectively.

## Description of *Roseovarius phycicola* sp. nov.

*Roseovarius phycicola* (phy.ci′co.la. L. n. *phycos*, seaweed; L. suffix. *-cola* (from L. masc./fem. n. *incola*), an inhabitant of a place; N.L. masc. n. *phycicola*, an inhabitant of seaweed).

Colonies on MA are circular, convex, smooth and greyish yellow in colour. Cells are Gram-stain-negative, strictly aerobic, and non-motile rods (0.7–1.0 µm in width and 1.0–1.6 µm in length), demonstrating oxidase- and catalase-positive activities. Growth occurs at 20–30 °C (optimum, 25 °C) and pH 6.0–9.0 (optimum, pH 8.0) and in the presence of 2.0–5.0% (w/v) NaCl (optimum, 3.0%). Nitrate reduction, indole production and d-glucose fermentation are negative. Aesculin is hydrolysed, but casein, Tween 20, Tween 80, tyrosine, starch and gelatin are not. Urease and arginine dihydrolase activities are negative. Assimilation of *N*-acetylglucosamine, malic acid, d-glucose, l-arabinose, d-mannose, d-maltose, gluconate and adipic acid is positive, but assimilation of d-mannitol, phenylacetic acid, capric acid and citric acid is negative. Q-10 is the sole respiratory quinone, and major cellular fatty acids (>5%) are summed feature 8 (comprising C_18 : 1_* ω7*c and/or C_18 : 1_* ω6*c), C_16 : 0_ and C_16 : 0_-2OH. PG, PC, an AL, a PL and two Ls are detected as major polar lipids.

The type strain is S88^T^ (=KACC 23423^T^ =JCM 36647^T^), isolated from a marine red alga *L. hakodatensis*. The total genome size and DNA G+C content calculated from the whole-genome (a chromosome and a plasmid) sequence of the type strain are 3637 kb and 57.0%, respectively. The GenBank accession numbers of the 16S rRNA gene and genome sequences of S88^T^ are OR431746 and CP146069–CP146070, respectively.

## Description of *Roseovarius rhodophyticola* sp. nov.

*Roseovarius rhodophyticola* (rho.do.phy.ti′co.la. N.L. neut. pl. n. *Rhodophyta*, the division of the red algae; L. suffix. -*cola* (from L. masc./fem. n. *incola*), an inhabitant of a place; N.L. masc. n. *rhodophyticola*, inhabitant of *Rhodophyta*).

Colonies on MA are circular, convex, smooth and greyish yellow in colour. Cells are Gram-stain-negative, strictly aerobic and non-motile rods (0.6–0.7 µm in width and 0.9–2.0 µm in length), demonstrating oxidase- and catalase-positive activities. Growth occurs at 20–30 °C (optimum, 25 °C) and pH 7.0–9.0 (optimum, pH 8.0) and in the presence of 2.0–5.0% (w/v) NaCl (optimum, 3.0%). Nitrate reduction, indole production and d-glucose fermentation are negative. Aesculin is hydrolysed, but casein, Tween 20, Tween 80, tyrosine, starch and gelatin are not. Urease and arginine dihydrolase activities are negative. Assimilation of l-arabinose, d-maltose, citric acid, phenylacetic acid and adipic acid is positive, but assimilation of d-glucose, d-mannitol, d-mannose, gluconate, *N*-acetylglucosamine, malic acid and capric acid is negative. Q-10 is the sole respiratory quinone, and major cellular fatty acids (>5%) are summed feature 8 (comprising C_18 : 1_* ω7*c and/or C_18 : 1_* ω6*c) and C_16 : 0_. PG, PC, an AL, a PL and an L are detected as major polar lipids.

The type strain is W115^T^ =KACC 23690^T^ =JCM 36651^T^), isolated from a marine red alga *S. dubyi*. The total genome size and DNA G+C content calculated from the whole genome (a chromosome and two plasmids) sequence of the type strain are 3717 kb and 56.5%, respectively. The GenBank accession numbers of the 16S rRNA gene and genome sequences of strain W115^T^ are OR431747 and CP146606–CP146607, respectively.

## supplementary material

10.1099/ijsem.0.006574 Uncited Fig. S1.

## Abbreviations

ANI: average nucleotide identity
ASW: artificial seawater
CAZy: Carbohydrate-Active enZyme
dDDH: digital DNA–DNA hybridization
GTDB-Tk: Genome Taxonomy Database Toolkit
LB: Luria-Bertani
MA: marine agar
MB: marine broth
ML: maximum-likelihood
MP: maximum-parsimony
NA: nutrient agar
NJ: neighbour-joining
PC: phosphatidylcholine
PG: phosphatidylglycerol
Q-10: ubiquinone-10
R2A: Reasoner’s 2A
TSA: tryptic soy agar
