# Supplementary material for: Roseovarius phycicola sp. nov. and Roseovarius rhodophyticola sp. nov., isolated from marine red algae
Source: Int J Syst Evol Microbiol. 2024 Nov 11;74(11):006574. doi: 10.1099/ijsem.0.006574 (PMC11554077; doi:10.1099/ijsem.0.006574)
Supplement: Uncited Fig. S1. [file ijsem-74-06574-s001.pdf]

Supplementary Information

Fig. S1. Maximum-likelihood (a) and maximum-parsimony (b) trees showing the phylogenetic relationships of strains S88^T and W115^T and their closely related taxa, based on 16S rRNA gene sequences. Bootstrap values exceeding 70% are presented on nodes as percentages from 1000 replicates. *Tropicimonas sediminicola* DSM 29339^T (JF748735) was employed as an outgroup. Scale bars in panels A and B represent substitutions per nucleotide and nucleotide substitutions over the entire sequences, respectively.

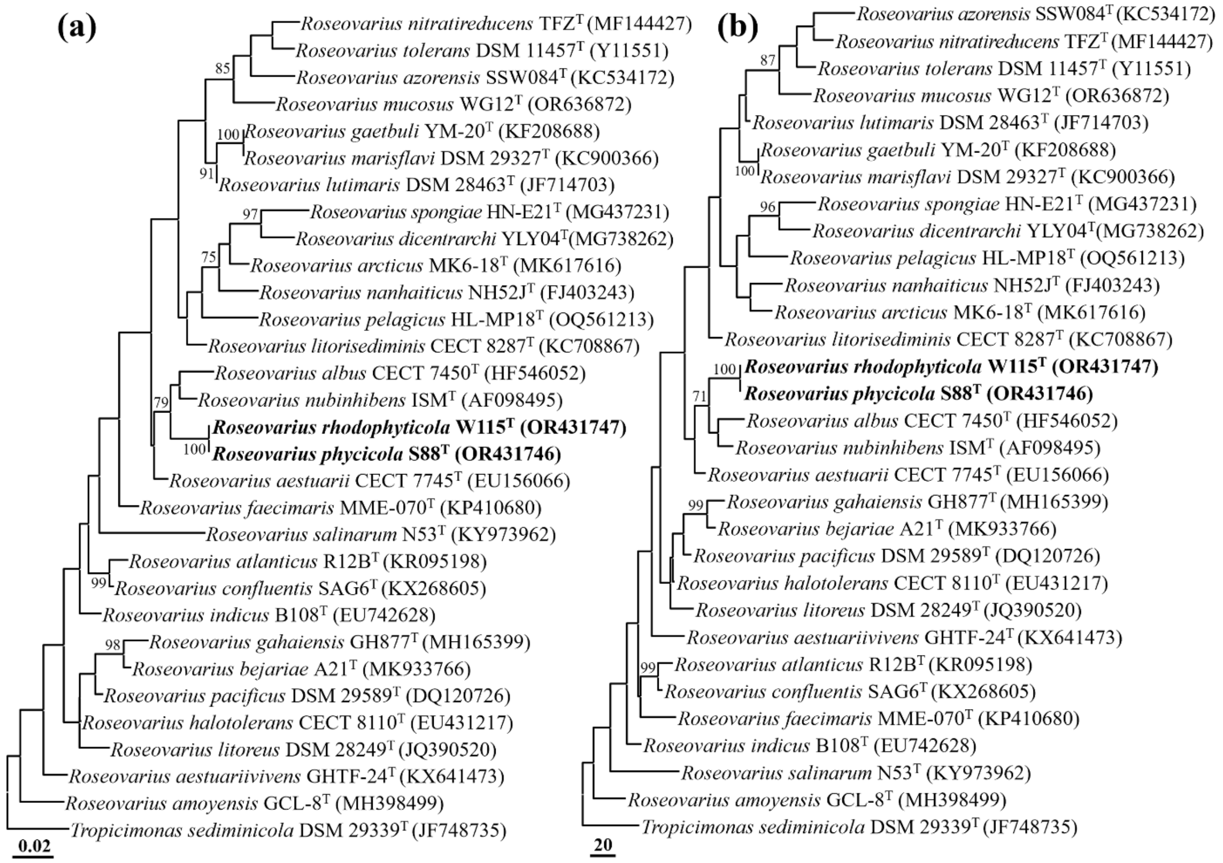

Fig. S2. Transmission electron micrographs of negatively stained (using 2% (w/v) uranyl acetate) cells cultivated on marine agar at 25°C for 3 days showing the general morphologies of strains S88^T (A) and W115^T (B). Scale bars, 0.5 μm.

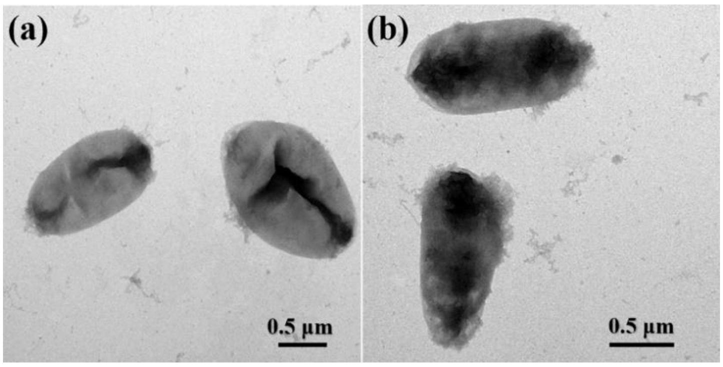

Fig. S3. Two-dimensional thin-layer chromatograms (TLC) showing the polar lipid profiles of strains S88^T and W115^T. Solvent systems: (I) chloroform-methanol-water (65:25:4, v/v/v) and (II) chloroform-acetic acid-methanol-water (80:15:12:4, v/v/v/v). The TLC plates were sprayed with 10% ethanolic molybdophosphoric acid (a), ninhydrin (b), Dittmer-Lester (c), and α-naphthol/sulfuric acid (d) reagents for the detection of total polar lipids, aminolipids, phospholipids, and glycolipids, respectively. PG, phosphatidylglycerol; PC, phosphatidylcholine; PL, unidentified phospholipid; AL, unidentified aminolipid; L, unidentified polar lipid.

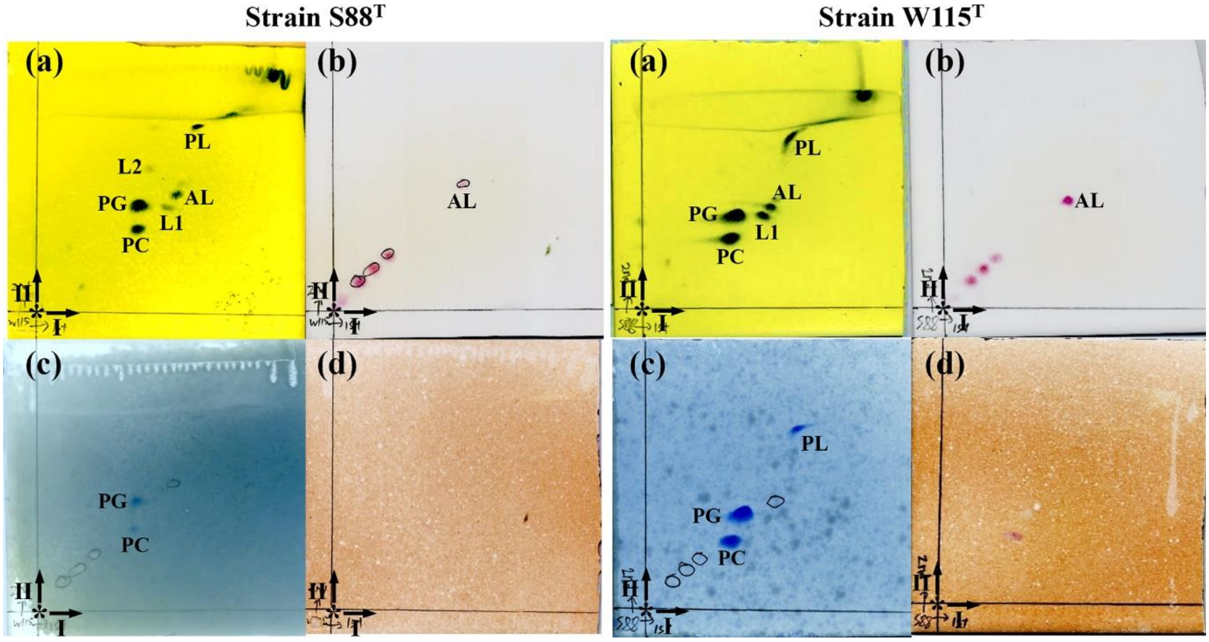

Table S1. Genome relatedness among strains S88^T and W115^T and closely related type strains of the genus *Roseiovarius*

Taxa: 1, strain S88^T (CP146069-CP146070); 2, strain W115^T (CP146606-CP146607); 3, *R. nubinhibens* ISM^T (AALY00000000); 4, *R. albus* CECT 7450^T (FWFX00000000); 5, *R. faecimaris* MME-070^T (CP034347-CP034348); 6, *R. aestuariivivens* GHTF-24^T (SJEY00000000); 7, *R. halotolerans* DSM 29507^T (RBXI00000000).
†ANI, average nucleotide identity; dDDH, digital DNA-DNA hybridization.

		dDDH† value (%)						
		**1**	**2**	**3**	**4**	**5**	**6**	**7**
ANI† value (%)	**1**	–	36.8	20.3	18.5	19.2	19.0	19.2
	**2**	88.8	–	18.8	19.0	19.3	18.8	18.1
	**3**	72.6	72.2	–	18.7	19.9	19.3	20.0
	**4**	71.3	71.3	71.9	–	18.3	17.7	18.3
	**5**	73.0	73.0	75.0	72.1	–	19.3	19.3
	**6**	73.3	73.7	74.0	71.0	74.2	–	19.1
	**7**	72.8	72.6	75.4	71.9	75.3	74.9	–

Table S2. Cellular fatty acid compositions (%) of strains S88^T and W115^T and closely related type strains of the genus *Roseovarius*

Taxa: 1, strain S88^T (this study); 2, strain W115^T (this study); 3, *R. nubinhibens* DSM 15170^T; 4, *R. albus* KCTC 22653^T; 5, *R. faecimaris* KCCM 43142^T; 6, *R. aestuariivivens* KCTC 52454^T; 7, *R. halotolerans* KCTC 22224^T. All data were obtained from this study. Data are expressed as percentages of the total fatty acids, and fatty acids constituting less than 1.0% in all strains are not shown. Major components (>5.0%) are highlighted in bold. Symbols: tr, trace amount (<1.0%); –, not detected.

**Fatty acid**	**1**	**2**	**3**	**4**	**5**	**6**	**7**
Saturated:							
$C_{10:0}$	2.8	1.5	4.9	**7.0**	–	–	2.4
$C_{12:0}$	4.9	2.2	4.7	**5.2**	–	3.2	3.9
$C_{14:0}$	tr	tr	–	–	–	1.0	tr
$C_{16:0}$	**10.4**	**16.4**	**13.3**	**6.5**	**5.0**	**10.2**	**8.7**
$C_{17:0}$	tr	tr	tr	tr	3.54	tr	tr
$C_{18:0}$	2.8	4.3	1.0	Tr	3.5	3.2	2.2
Unsaturated:							
iso-$C_{15:1}$ $\omega 9c$	–	–	1.3	1.1	–	–	–
iso-$C_{17:1}$ $\omega 5c$	tr	tr	tr	tr	–	1.0	tr
$C_{18:1}$ $\omega 7c$ 11-methyl	4.9	–	tr	tr	**6.1**	2.4	**7.5**
cyclo-$C_{19:0}$ $\omega 8c$	2.5	3.0	**12.6**	3.9	–	–	**5.1**
$C_{20:1}$ $\omega 7c$	tr	–	tr	tr	1.3	1.1	tr
Branched:							
iso-$C_{12:0}$	–		1.2	1.1	–	–	–
iso-$C_{17:0}$	tr	tr	1.1	tr	–	–	tr
iso-$C_{19:0}$	–	tr	–	–	1.1	2.9	tr
anteiso-$C_{15:0}$	tr	tr	1.4	1.3	tr	tr	tr
anteiso-$C_{17:0}$	tr	tr	1.2	1.2	1.1	2.9	tr
Hydroxy:							
$C_{11:0}$ 2-OH	–	–	1.3	1.2	–	–	–
$C_{12:0}$ 2-OH	–	1.9	–	–	–	–	–
$C_{12:0}$ 3-OH	4.2	3.6	1.7	–	–	1.8	2.4
$C_{12:1}$ 3-OH	–	–	–	–	–	–	1.8
$C_{15:0}$ 2-OH	tr	tr	–	–	1.3	1.1	tr
$C_{16:0}$ 2-OH	**5.6**	–	–	**5.0**	**5.0**	–	tr
Summed feature*:							
2	–	–	–	3.3	–	–	–
3	tr	tr	2.0	tr	tr	tr	tr
6	tr	tr	–	–	–	1.2	tr
7	tr	tr	1.0	1.0	1.7	tr	tr
8	**52.2**	**60.4**	**42.0**	**50.9**	**68.7**	**63.9**	**55.7**

*Summed Features are fatty acids that cannot be resolved reliably from another fatty acid using the chromatographic conditions chosen. The MIDI system groups these fatty acids together as one feature with a single percentage of the total. Summed feature 2, $C_{12:0}$ aldehyde; summed feature 3, $C_{16:1}$ $\omega 7c$ and/or $C_{16:1}$ $\omega 6c$; summed feature 6, $C_{19:1}$ $\omega 11c$ and/or $C_{19:1}$ $\omega 9c$; summed feature 7, $C_{19:1}$ $\omega 7c$ and/or $C_{19:1}$ $\omega 6c$; summed feature 8, $C_{18:1}$ $\omega 7c$ and/or $C_{18:1}$ $\omega 6c$.
